# Relationship between red cell distribution width/albumin ratio and carotid plaque in different glucose metabolic states in patients with coronary heart disease: a RCSCD-TCM study in China

**DOI:** 10.1186/s12933-023-01768-w

**Published:** 2023-02-22

**Authors:** Mengnan Huang, Fanfan Liu, Zhu Li, Yijia Liu, Jinyu Su, Mei Ma, Yuanyuan He, Huaien Bu, Shan Gao, Hongwu Wang, Chunquan Yu

**Affiliations:** Tianjin University of Traditional Chinese Medicine, 10 Poyanghu Road, West Area, Tuanbo New Town, Jinghai District, Tianjin, 301617 China

**Keywords:** Coronary heart disease, Red cell distribution width, Albumin, RDW/ALB ratio, Carotid plaque, Glucose metabolism states

## Abstract

**Background:**

Red cell distribution width/albumin ratio (RAR) is thought to be associated with the prognosis of a variety of diseases, including diabetes and heart failure. To date, no studies have focused on the relationship between RAR and carotid plaque in patients with coronary heart disease (CHD).

**Methods:**

A total of 10,267 patients with CHD were divided according to RAR quartiles (Q1: RAR ≤ 2.960; Q2: 2.960 < RAR ≤ 3.185; Q3: 3.185 < RAR < 3.441; Q4: RAR ≥ 3.441). Logistic regression was used to analyze the relationship between RAR and carotid plaques in CHD patients. The relationship between RAR and carotid plaques in according to sex, age and glucose regulation state groups were also assessed.

**Results:**

Among the 10,267 participants, 75.43% had carotid plaques. After adjusting for confounding factors, RAR was found to be associated with carotid plaque formation (OR: 1.23; 95% CI 1.08–1.39). The risk of carotid plaque formation in the Q4 group was 1.24 times higher than that in the Q1 group. After multivariate adjustment, RAR was associated with the risk of carotid plaque in female (OR: 1.29; 95% CI 1.09–1.52). And the relationship between RAR and carotid plaques in patients younger than 60 years old (OR: 1.43; 95% CI 1.16–1.75) was stronger than that in those older than 60 years old (OR: 1.29; 95% CI 1.10–1.51). Under different glucose metabolism states, RAR had the highest correlation with the risk of carotid plaques in diabetes patients (OR: 1.28; 95% CI 1.04–1.58).

**Conclusions:**

RAR was significantly related to carotid plaques in patients with CHD. In addition, the correlation between RAR and the incidence of carotid plaque in patients with CHD was higher in women and middle-aged and elderly patients. In patients with CHD and diabetes, the correlation between RAR and carotid plaque was higher.

**Graphical Abstract:**

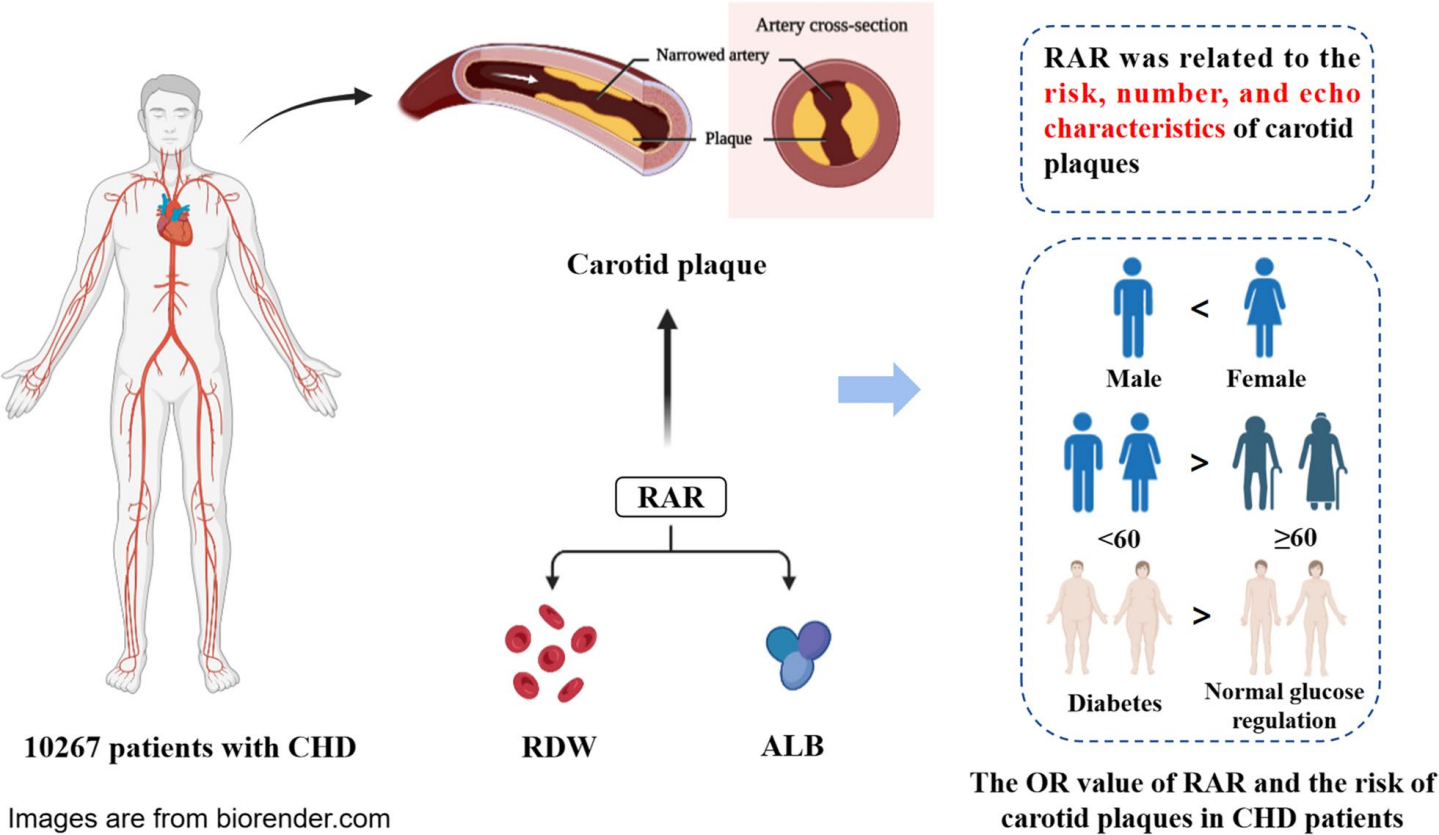

**Supplementary Information:**

The online version contains supplementary material available at 10.1186/s12933-023-01768-w.

## Introduction

Coronary heart disease (CHD) is a common disease of the cardiovascular system. Its pathogenesis is caused by coronary artery atherosclerosis, which causes coronary artery stenosis or occlusion, hinders the blood and oxygen supply of myocardial tissue, and then causes the necrosis of myocardial tissue, leading to the occurrence of the disease. Coronary atherosclerosis is a risk factor for CHD. Carotid plaque is related to coronary atherosclerotic lesions, and carotid atherosclerotic plaques are similar to coronary atherosclerotic plaques in terms of pathogenesis and pathophysiological basis [1]. Studies have found that the existence of carotid plaque is independently related to the risk of CHD [2]. Increased carotid plaque and carotid intima-media thickness (IMT) may increase the risk of cardiovascular disease (CVD) [3]. The condition of carotid atherosclerosis may indicate the occurrence and development of CHD.

Red cell distribution width (RDW) is a simple and readily available parameter that represents the heterogeneity of red cell volume and has traditionally been used in the differential diagnosis of anemia. In recent years, RDW has been found to be associated with a variety of disease processes and prognosis, such as CVD, venous thromboembolism, etc. [4]. RDW has prognostic value for heart failure and CHD, and is an independent risk factor for frailty in elderly patients with CHD [5, 6]. And RDW is associated with a higher risk of developing diabetes [7]. Some researchers believe that chronic hyperglycaemia may mediate the relationship between high RDW and cardiovascular disease [8], RDW may be a useful clinical marker of vascular complications in diabetes (DM) [9]. Serum albumin (ALB) is a biochemical marker of nutritional status [10]. ALB level is strongly negatively correlated with the risk of death from CVD, as well as with the reduction of anti-inflammatory activity and oxidative stress [11]. RDW/ALB ratio (RAR) is a new combination parameter that can be quickly and easily obtained during laboratory tests on admission and can be used in a variety of clinical environments. Studies have shown that RAR is closely associated with CHD, and in addition, RAR showed a better predictive effect than RDW or albumin alone [12, 13].

No studies have shown the relationship between RAR and carotid plaque in patients with CHD. In this study, we sought to explore the impact of RAR on carotid plaque and clarify the association between RAR and carotid plaque in the different glucose metabolic statuses of CHD patients.

## Methods

### Study participants

**Fig. 1:**
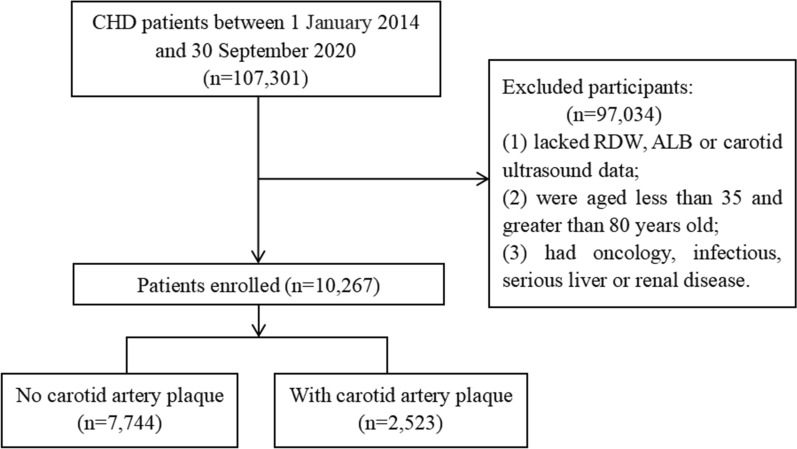
Flowchart of case recruitment

This large-scale, multi-center, retrospective, cross-sectional study included 107,301 CHD patients hospitalized in 6 hospitals in Tianjin from January 1, 2014 to September 30, 2020 [14]. This study excluded patients younger than 35 years or older than 80 years old, patients with tumor, infectious disease, or severe liver or kidney disease, and patients lacking data on RDW, ALB, and carotid ultrasound measurements. A total of 10,267 participants were included in the study. A flowchart of the patients recruitment was shown in Fig. 1.

This study was approved by the ethics committee of Tianjin University of Traditional Chinese Medicine (TJUTCM-EC20190008) and registered with the Chinese Clinical Trial Registry (ChiCTR-1900024535) and ClinicalTrials.gov (NCT04026724).

### Data collection

Trained medical staff collected data of age, sex, smoking and drinking status, medical history, and prior medication history by means of a standard structured questionnaire [15, 16]. The systolic and diastolic blood pressures (SBP and DBP) were measured by experienced physicians using an electronic device. Hypertension was defined as SBP ≥ 130 mmHg or DBP ≥ 80 mmHg [17].

Fasting venous blood samples were taken from all participants on the second day of hospitalization. RDW, ALB, fasting plasma glucose (FPG), glycated hemoglobin (HbA1c), total cholesterol (TC), triglyceride (TG), high-density lipoprotein-cholesterol (HDL-C), and low-density lipoprotein-cholesterol (LDL-C) levels were measured using an automatic haematology analyzer. Standard laboratory procedure for quality control were strictly followed [18]. Hyperlipidemia was defined as: TC ≥ 6.2 mmol/L (240 mg/dL), TG ≥ 2.3 mmol/L (200 mg/dL) or LDL-C ≥ 4.1 mmol/L (160 mg/dL) or HDL-C ≤ 1.0 mmol/L (40 mg/dL) [19]. Normal glucose tolerance (NGT) was defined as FPG < 5.6 mmol/L or HbA1c < 5.7%, prediabetes (Pre-DM) was defined as FPG 5.6–6.9 mmol/L or HbA1c of 5.7–6.4%, DM was defined as FPG ≥ 7.0 mmol/L or HbA1c ≥ 6.5% [20].

Carotid ultrasound examinations were performed by trained and certified physicians using an ultrasound diagnostic system. In B-mode imaging, the common carotid artery, internal carotid artery, and carotid artery bifurcation were scanned and imaged. Carotid artery color-Doppler was analyzed by professional physicians according to the results of Doppler ultrasound, and the number of carotid plaque and echo characteristics were recorded. The number of carotid plaque was divided into single (n = 1) or multiple (n ≥ 2). The echo properties of carotid plaque can be divided into hypoechoic, isoechoic, hyperechoic, and mixed types. Implement strict quality control procedures for image acquisition and analysis, and inter-laboratory quality evaluations by certified personnel.

### Statistical analyses

The *χ*^2^ test and Kruskal–Wallis H test were used to compare the characteristics of the participants in the different groups. Odds ratios (ORs) and 95% confidence intervals (CIs) of carotid plaques were estimated for RAR using logistic regression. Four logistic regression models were constructed: Model a, unadjusted; Model b, adjusted for age, sex, SBP, DBP; Model c, adjusted for age, sex, SBP, DBP, TC, TG, smoking, drinking, use of antihypertensives, use of antilipidemic; and Model d was adjusted based on Model c with haematologic, rheumatical, diabetes, WBC, CRP, and Hb. The collinearity of the different models was tested before logistic regression. Missing values were imputed using the multiple imputation method. All statistical analyses were performed using SPSS 24.0 (IBM Corp, New York, NY, USA). *P* < 0.05 was considered statistically significant.

## Results

### Baseline characteristics

The baseline characteristics of participants are shown in Table 1. 10,267 participants were included for data analysis, the median age was 64 years old, and 5222 was female (50.86%). Among them, 7744 (75.43%) patients had carotid plaques. Based on the RAR quartering method, the participants were divided into four groups: Q1 (RAR ≤ 2.960), Q2 (2.960 < RAR ≤ 3.185), Q3 (3.185 < RAR < 3.441), and Q4 (RAR ≥ 3.441). There were differences in the risk, number, and echo characteristics of carotid plaques among the four RAR groups (*P* < 0.001). Compared with the Q1 group, the number of patients with carotid plaques in the Q4 group was higher. They were more likely to be older, and had lower levels of TC, TG, HDL-C and LDL-C.

**Table 1:** Characteristics of participants according to RAR quartile

Characteristic	Total (*N* = 10,267)	RAR quartile				*P* value
		Q1 (*n* = 2564)	Q2 (*n* = 2561)	Q3 (*n* = 2576)	Q4 (*n* = 2566)	
Sex, n (%)						0.033
Male	5045 (49.14)	1300 (50.70)	1206 (47.09)	1248 (48.45)	1291 (50.31)	
Female	5222 (50.86)	1264 (49.30)	1355 (52.91)	1328 (51.55)	1275 (49.69)	
Age (y)						
Total, median (IQR)	64 (59, 69)	62 (56, 67)	64 (58, 69)	65 (60, 70)	66 (61, 71)	< 0.001
< 60, n (%)	2856 (27.82)	970 (37.83)	768 (29.99)	611 (23.72)	507 (19.76)	< 0.001
≥ 60, n (%)	7411 (72.18)	1594 (62.17)	1793 (70.01)	1965 (76.28)	2059 (80.24)	
SBP, mmHg, median (IQR)	140 (128, 156)	140 (129, 157)	140 (129, 157)	140 (127, 157)	140 (125, 155)	0.002
DBP, mmHg, median (IQR)	83 (77, 90)	83 (79, 91)	83 (77, 91)	82 (77, 90)	80 (75, 90)	< 0.001
TC, mmol/L, median (IQR)	4.58 (3.81, 5.36)	4.82 (4.06, 5.58)	4.66 (3.91, 5.42)	4.53 (3.80, 5.25)	4.30 (3.52, 5.09)	< 0.001
TG, mmol/L, median (IQR)	1.44 (1.04, 2.05)	1.61 (1.14, 2.27)	1.52 (1.11, 2.10)	1.41 (1.03, 2.01)	1.26 (0.91, 1.77)	< 0.001
LDL-C, mmol/L, median (IQR)	2.76 (2.15, 3.42)	2.94 (2.31, 3.59)	2.82 (2.20, 3.47)	2.71 (2.13, 3.34)	2.57 (1.97, 3.25)	< 0.001
HDL-C, mmol/L, median (IQR)	1.06 (0.89, 1.26)	1.12 (0.95, 1.32)	1.07 (0.90, 1.27)	1.05 (0.89, 1.24)	1.01 (0.85, 1.20)	< 0.001
RDW-CV, (%), median (IQR)	12.70 (12.30, 13.30)	12.20 (11.90, 12.60)	12.60 (12.20, 12.90)	12.90 (12.50, 13.30)	13.50 (13.00, 14.20)	< 0.001
ALB, g/dL, median (IQR)	4.01 (3.78, 4.25)	4.40 (4.24, 4.58)	4.09 (3.97, 4.21)	3.89 (3.78, 4.03)	3.63 (3.45, 3.80)	< 0.001
RAR	3.19 (2.96, 3.44)	2.81 (2.71, 2.89)	3.07 (3.02, 3.13)	3.30 (3.24, 3.37)	3.68 (3.54, 3.99)	< 0.001
Smoking, n (%)	4432 (43.17)	1094 (42.67)	1069 (41.74)	1108 (43.01)	1161 (45.25)	0.074
Drinking, n (%)	3164 (30.82)	871 (33.97)	778 (30.38)	770 (29.89)	745 (29.03)	0.001
Hypertension, n (%)	7516 (73.21)	1941 (75.70)	1905 (74.39)	1848 (71.74)	1822 (71.01)	< 0.001
Hyperlipidemia, n (%)	3172 (30.90)	914 (35.65)	828 (32.33)	774 (30.05)	656 (25.57)	< 0.001
Use of antihypertensive, n (%)	7559 (73.62)	1862 (72.62)	1853 (72.35)	1919 (74.50)	1925 (75.02)	0.071
Use of antilipidemic, n (%)	7584 (73.87)	1968 (76.76)	1957 (76.42)	1917 (74.42)	1742 (67.89)	< 0.001
CIMT, mm, median (IQR)	0.10 (0.09, 0.12)	0.10 (0.09, 0.11)	0.10 (0.09, 0.12)	0.10 (0.09, 0.12)	0.10 (0.09, 0.12)	< 0.001
Carotid artery plaque, n (%)	7744 (75.43)	1829 (71.33)	1885 (73.60)	1954 (75.85)	2076 (80.90)	< 0.001
No. of carotid artery plaque, n (%)						< 0.001
0	2523 (24.57)	735 (28.67)	676 (26.40)	622 (24.15)	490 (19.10)	
1	421 (4.10)	132 (5.15)	118 (4.61)	79 (3.07)	92 (3.59)	
≥ 2	7323 (71.33)	1697 (66.19)	1767 (69.00)	1875 (72.79)	1984 (77.32)	
Carotid artery plaque echo property, n (%)						< 0.001
Hypoechoic plaque	512 (6.61)	124 (6.78)	130 (6.90)	135 (6.91)	123 (5.92)	
Isoechoic plaque	574 (7.41)	163 (8.91)	151 (8.01)	132 (6.76)	128 (6.17)	
Hyperechoic plaque	4312 (55.68)	1048 (57.30)	1062 (56.34)	1080 (55.27)	1122 (54.05)	
Mixture plaque	2346 (30.29)	494 (27.01)	542 (28.75)	607 (31.06)	703 (33.86)	

### Relationship between RAR and the risk of carotid plaques

As shown in Table 2, when used as a continuous variable, after adjusting for confounding factors, RAR was significantly associated with the risk of carotid plaque (OR: 1.23; 95% CI 1.08–1.39). When RAR as a categorical variable, the risk of carotid plaque in the Q4 group was 1.24 times higher than that in the Q1 group. In further analysis, in the unadjusted or adjusted model, the P_trend_ of RAR and carotid plaques was consistent with the results of RAR as a continuous variable (*P* < 0.01).

**Table 2:** Relationship between the RAR and the risk of carotid plaques

Variables	Carotid artery plaques							
	OR (95% CI)^a^	*P*-value	OR (95% CI)^b^	*P*-value	OR (95% CI)^c^	*P*-value	OR (95% CI)^d^	*P*-value
RAR	1.52 (1.37, 1.69)	< 0.001	1.22 (1.09, 1.36)	< 0.001	1.25 (1.12, 1.40)	< 0.001	1.23 (1.08, 1.39)	0.001
Q1	Reference		Reference		Reference		Reference	
Q2	1.12 (0.99, 1.27)	0.069	0.98 (0.85, 1.11)	0.706	0.98 (0.86, 1.12)	0.747	0.97 (0.85, 1.12)	0.689
Q3	1.26 (1.12, 1.43)	< 0.001	0.98 (0.86, 1.13)	0.804	0.99 (0.86, 1.14)	0.876	0.96 (0.84, 1.11)	0.591
Q4	1.70 (1.49, 1.94)	< 0.001	1.25 (1.08, 1.44)	0.003	1.28 (1.10, 1.48)	0.001	1.24 (1.07, 1.45)	0.006
*P*-trend		< 0.001		0.003		0.001		0.008

^a^Model1: unadjusted

^b^Model2: adjusted for sex, age, SBP, DBP

^c^Model3: adjusted for sex, age, SBP, DBP, TC, TG, smoking, drinking, use of antihypertensives, use of antilipidemic

^d^Model4: adjusted for sex, age, SBP, DBP, TC, TG, smoking, drinking, use of antihypertensives, use of antilipidemic, haematologic, rheumatical, diabetes, WBC, CRP, Hb

### Relationship between RAR and carotid plaques based on sex and age

As shown in Tables 3 and 4, we observed that there was a significant relationship between RAR and carotid plaque in different sex or age stratification. After multivariate adjustment, RAR was associated with the risk of carotid plaque in female (OR: 1.29; 95% CI 1.09–1.52). After multivariate adjustment, the relationship between RAR and carotid plaques in patients younger than 60 years old (OR: 1.43; 95% CI 1.16–1.75) was stronger than that in those older than 60 years old (OR: 1.29; 95% CI 1.10–1.51). Regardless of sex or age, with Q1 as a reference, Q4 was significantly associated with an increased risk of carotid plaque, which was still significant after multifactorial adjustment.

**Table 3:** Relationship between the RAR and the risk of carotid plaques according to sex

Sex	Variables	Carotid artery plaques							
		OR (95% CI)^a^	*P*-value	OR (95% CI)^b^	*P*-value	OR (95% CI)^c^	*P*-value	OR (95% CI)^d^	*P*-value
Male	RAR	1.59 (1.34, 1.88)	< 0.001	1.16 (0.98, 1.36)	0.082	1.19 (1.01, 1.40)	0.044	1.16 (0.96, 1.40)	0.133
	Q1	Reference		Reference		Reference		Reference	
	Q2	1.30 (1.07, 1.59)	0.010	1.07 (0.86, 1.32)	0.546	1.07 (0.87, 1.33)	0.512	1.07 (0.86, 1.33)	0.553
	Q3	1.38 (1.13, 1.68)	0.002	0.99 (0.80, 1.23)	0.945	0.99 (0.80, 1.23)	0.947	0.97 (0.78, 1.21)	0.816
	Q4	1.94 (1.57, 2.39)	< 0.001	1.24 (0.99, 1.56)	0.060	1.29 (1.02, 1.62)	0.035	1.27 (0.99, 1.63)	0.064
Female	RAR	1.49 (1.30, 1.72)	< 0.001	1.27 (1.10, 1.47)	0.001	1.31 (1.12, 1.52)	0.001	1.29 (1.09, 1.52)	0.002
	Q1	Reference		Reference		Reference		Reference	
	Q2	1.07 (0.91, 1.26)	0.391	0.92 (0.77, 1.09)	0.345	0.92 (0.77, 1.10)	0.348	0.91 (0.77, 1.09)	0.313
	Q3	1.24 (1.05, 1.46)	0.011	0.98 (0.82, 1.17)	0.797	0.98 (0.82, 1.18)	0.861	0.95 (0.79, 1.14)	0.569
	Q4	1.61 (1.36, 1.91)	< 0.001	1.25 (1.04, 1.50)	0.020	1.27 (1.05, 1.53)	0.014	1.23 (1.01, 1.50)	0.042

^a^Model1: unadjusted

^b^Model2: adjusted for age, SBP, DBP

^c^Model3: adjusted for age, SBP, DBP, TC, TG, smoking, drinking, use of antihypertensives, use of antilipidemic

^d^Model4: adjusted for age, SBP, DBP, TC, TG, smoking, drinking, use of antihypertensives, use of antilipidemic, haematologic, rheumatical, diabetes, WBC, CRP, Hb

**Table 4:** Relationship between the RAR and the risk of carotid plaques according to age

Age	Variables	Carotid artery plaques							
		OR (95% CI)^a^	*P*-value	OR (95% CI)^b^	*P*-value	OR (95% CI)^c^	*P*-value	OR (95% CI)^d^	*P*-value
< 60	RAR	1.30 (1.11, 1.53)	0.001	1.36 (1.14, 1.62)	0.001	1.43 (1.20, 1.71)	< 0.001	1.43 (1.16, 1.75)	0.001
	Q1	Reference		Reference		Reference		Reference	
	Q2	1.12 (0.93, 1.36)	0.244	1.13 (0.92, 1.39)	0.236	1.13 (0.92, 1.39)	0.245	1.13 (0.92, 1.40)	0.248
	Q3	0.99 (0.81, 1.21)	0.908	1.01 (0.81, 1.25)	0.940	1.04 (0.83, 1.29)	0.747	1.01 (0.81, 1.26)	0.950
	Q4	1.43 (1.15, 1.79)	0.002	1.51 (1.19, 1.91)	0.001	1.61 (1.26, 2.05)	< 0.001	1.60 (1.23, 2.08)	< 0.001
≥ 60	RAR	1.35 (1.18, 1.54)	< 0.001	1.32 (1.15, 1.52)	< 0.001	1.34 (1.16, 1.54)	< 0.001	1.29 (1.10, 1.51)	0.002
	Q1	Reference		Reference		Reference		Reference	
	Q2	0.96 (0.81, 1.14)	0.662	1.00 (0.84, 1.19)	0.982	1.00 (0.84, 1.19)	0.960	1.00 (0.84, 1.19)	0.967
	Q3	1.11 (0.94, 1.32)	0.215	1.13 (0.95, 1.34)	0.182	1.11 (0.94, 1.33)	0.228	1.09 (0.92, 1.31)	0.317
	Q4	1.37 (1.15, 1.63)	< 0.001	1.37 (1.15, 1.64)	< 0.001	1.38 (1.15, 1.65)	0.001	1.33 (1.10, 1.60)	0.003

^a^Model1: unadjusted

^b^Model2: adjusted for sex, SBP, DBP

^c^Model3: adjusted for sex, SBP, DBP, TC, TG, smoking, drinking, use of antihypertensives, use of antilipidemic

^d^Model4: adjusted for sex, SBP, DBP, TC, TG, smoking, drinking, use of antihypertensives, use of antilipidemic, haematologic, rheumatical, diabetes, WBC, CRP, Hb

### Relationship between the RAR and the risk of carotid plaques according to glucose regulation state

As shown in Table 5, after multivariate adjustment, under different glucose metabolism states, RAR had the highest correlation with the risk of carotid plaques in DM status (OR: 1.28; 95% CI 1.04–1.58). Taking the Q1 as a reference, Q4 was associated with an increased risk of carotid plaques in diabetes. In joint effect analyses, there was a significant additive interaction between RAR and diabetes on the risk of carotid plaque in patients with CHD (RERI 0.337; 95% CI 0.142, 0.532; AP 0.183; 95% CI 0.077, 0.290; SI 1.668; 95% CI 1.107, 2.514) (Additional file 1: Table S1).

**Table 5:** Relationship between the RAR and the risk of carotid plaques according to glucose regulation state

Glucose regulation state	Variables	Carotid artery plaques							
		OR (95% CI)^a^	*P*-value	OR (95% CI)^b^	*P*-value	OR (95% CI)^c^	*P*-value	OR (95% CI)^d^	*P*-value
Normal glucose regulation	RAR	1.68 (1.40, 2.00)	< 0.001	1.23 (1.02, 1.49)	0.030	1.23 (1.02, 1.49)	0.033	1.26 (1.02, 1.56)	0.032
	Q1	Reference		Reference		Reference		Reference	
	Q2	1.16 (0.95, 1.42)	0.138	0.99 (0.79, 1.24)	0.910	0.99 (0.79, 1.24)	0.934	0.97 (0.77, 1.22)	0.806
	Q3	1.34 (1.09, 1.65)	0.006	0.99 (0.79, 1.25)	0.929	0.98 (0.77, 1.24)	0.845	0.95 (0.75, 1.21)	0.671
	Q4	1.91 (1.54, 2.37)	< 0.001	1.32 (1.04, 1.68)	0.024	1.31 (1.02, 1.67)	0.035	1.33 (1.02, 1.72)	0.034
Prediabetes	RAR	1.34 (1.11, 1.63)	0.002	1.07 (0.88, 1.31)	0.487	1.09 (0.89, 1.33)	0.409	1.11 (0.88, 1.40)	0.368
	Q1	Reference		Reference		Reference		Reference	
	Q2	1.18 (0.94, 1.48)	0.143	1.04 (0.82, 1.34)	0.736	1.06 (0.82, 1.35)	0.669	1.05 (0.82, 1.35)	0.699
	Q3	1.38 (1.10, 1.74)	0.006	1.05 (0.81, 1.35)	0.718	1.03 (0.80, 1.34)	0.810	1.05 (0.81, 1.37)	0.697
	Q4	1.48 (1.17, 1.87)	0.001	1.07 (0.82, 1.39)	0.612	1.09 (0.83, 1.42)	0.541	1.12 (0.84, 1.49)	0.453
Diabetes	RAR	1.53 (1.28, 1.84)	< 0.001	1.31 (1.09, 1.58)	0.004	1.41 (1.16, 1.70)	< 0.001	1.28 (1.04, 1.58)	0.021
	Q1	Reference		Reference		Reference		Reference	
	Q2	1.06 (0.85, 1.31)	0.614	0.93 (0.74, 1.17)	0.553	0.94 (0.74, 1.18)	0.586	0.92 (0.73, 1.16)	0.488
	Q3	1.11 (0.89, 1.37)	0.356	0.92 (0.73, 1.15)	0.449	0.96 (0.76, 1.20)	0.697	0.91 (0.72, 1.15)	0.442
	Q4	1.71 (1.35, 2.15)	< 0.001	1.32 (1.03, 1.69)	0.029	1.42 (1.10, 1.82)	0.007	1.27 (0.97, 1.66)	0.079

^a^Model1: unadjusted

^b^Model2: adjusted for sex, age, SBP, DBP

^c^Model3: adjusted for sex, age, SBP, DBP, TC, TG, smoking, drinking, use of antihypertensives, use of antilipidemic

^d^Model4: adjusted for sex, age, SBP, DBP, TC, TG, smoking, drinking, use of antihypertensives, use of antilipidemic, haematologic, rheumatical, WBC, CRP, Hb

In addition, we also observed the relationship between age and sex of RAR and the risk of carotid plaque under different glucose metabolism states. RAR was associated with the risk of carotid plaque in female with DM status (OR: 1.33; 95% CI 1.01–1.76) (Table 6). The association in patients younger than 60 years old (OR: 1.59; 95% CI 1.13–2.24) with DM status was stronger than that in those older than 60 years old (OR: 1.35; 95% CI 1.03–1.76) (Table 7).

**Table 6:** Relationship between RAR and the risk of carotid plaques according to different glucose regulation state and sex

Glucose regulation state	Sex	Variables	Carotid artery plaques							
			OR (95% CI)^a^	*P*-value	OR (95% CI)^b^	*P*-value	OR (95% CI)^c^	*P*-value	OR (95% CI)^d^	*P*-value
Normal glucose regulation	Male	RAR	1.62 (1.23, 2.13)	0.001	1.17 (0.89, 1.53)	0.266	1.22 (0.92, 1.61)	0.169	1.25 (0.91, 1.71)	0.167
		Q1	Reference		Reference		Reference		Reference	
		Q2	1.40 (1.01, 1.93)	0.045	1.18 (0.83, 1.67)	0.364	1.21 (0.85, 1.73)	0.293	1.16 (0.81, 1.66)	0.426
		Q3	1.61 (1.16, 2.24)	0.005	1.18 (0.83, 1.69)	0.365	1.20 (0.83, 1.72)	0.334	1.14 (0.79, 1.64)	0.495
		Q4	2.22 (1.57, 3.13)	< 0.001	1.45 (1.00, 2.11)	0.051	1.52 (1.04, 2.23)	0.031	1.54 (1.02, 2.31)	0.038
	Female	RAR	1.62 (1.27, 2.07)	< 0.001	1.30 (1.00, 1.68)	0.048	1.26 (0.97, 1.65)	0.085	1.30 (0.98, 1.74)	0.074
		Q1	Reference		Reference		Reference		Reference	
		Q2	1.07 (0.82, 1.39)	0.619	0.87 (0.65, 1.17)	0.359	0.85 (0.64, 1.14)	0.283	0.84 (0.62, 1.13)	0.248
		Q3	1.14 (0.87, 1.50)	0.345	0.87 (0.64, 1.18)	0.380	0.84 (0.62, 1.15)	0.272	0.82 (0.60, 1.13)	0.223
		Q4	1.69 (1.27, 2.24)	< 0.001	1.24 (0.90, 1.69)	0.188	1.17 (0.85, 1.62)	0.335	1.20 (0.85, 1.68)	0.302
Prediabetes	Male	RAR	1.46 (1.07, 1.99)	0.018	1.03 (0.76, 1.39)	0.856	1.01 (0.74, 1.37)	0.968	0.98 (0.68, 1.41)	0.922
		Q1	Reference		Reference		Reference		Reference	
		Q2	1.37 (0.94, 2.00)	0.101	1.11 (0.74, 1.68)	0.604	1.13 (0.74, 1.71)	0.572	1.12 (0.74, 1.71)	0.594
		Q3	1.35 (0.93, 1.96)	0.110	0.85 (0.57, 1.29)	0.453	0.82 (0.54, 1.24)	0.344	0.82 (0.54, 1.25)	0.357
		Q4	1.58 (1.07, 2.33)	0.021	0.91 (0.60, 1.40)	0.679	0.89 (0.57, 1.38)	0.604	0.89 (0.55, 1.45)	0.641
	Female	RAR	1.29 (1.00, 1.66)	0.047	1.11 (0.85, 1.44)	0.444	1.17 (0.89, 1.53)	0.253	1.24 (0.92, 1.67)	0.154
		Q1	Reference		Reference		Reference		Reference	
		Q2	1.18 (0.88, 1.58)	0.260	1.02 (0.75, 1.39)	0.902	1.03 (0.76, 1.42)	0.837	1.03 (0.75, 1.41)	0.868
		Q3	1.48 (1.09, 2.00)	0.012	1.18 (0.86, 1.64)	0.310	1.19 (0.86, 1.65)	0.302	1.24 (0.89, 1.73)	0.211
		Q4	1.51 (1.11, 2.05)	0.009	1.17 (0.84, 1.63)	0.360	1.23 (0.88, 1.74)	0.230	1.27 (0.89, 1.82)	0.184
Diabetes	Male	RAR	1.70 (1.27, 2.29)	< 0.001	1.29 (0.97, 1.73)	0.080	1.40 (1.04, 1.89)	0.025	1.25 (0.90, 1.75)	0.182
		Q1	Reference		Reference		Reference		Reference	
		Q2	1.20 (0.85, 1.68)	0.296	0.98 (0.69, 1.40)	0.909	0.97 (0.68, 1.39)	0.875	0.95 (0.67, 1.37)	0.792
		Q3	1.22 (0.87, 1.71)	0.249	0.95 (0.66, 1.35)	0.760	0.98 (0.68, 1.41)	0.915	0.94 (0.65, 1.36)	0.748
		Q4	2.04 (1.41, 2.97)	< 0.001	1.39 (0.94, 2.07)	0.100	1.57 (1.05, 2.36)	0.029	1.41 (0.91, 2.18)	0.122
	Female	RAR	1.49 (1.18, 1.90)	0.001	1.34 (1.05, 1.71)	0.019	1.42 (1.10, 1.83)	0.007	1.33 (1.01, 1.76)	0.041
		Q1	Reference		Reference		Reference		Reference	
		Q2	1.01 (0.76, 1.35)	0.940	0.91 (0.67, 1.23)	0.527	0.91 (0.67, 1.24)	0.547	0.89 (0.65, 1.22)	0.471
		Q3	1.11 (0.84, 1.47)	0.452	0.90 (0.67, 1.21)	0.483	0.94 (0.69, 1.27)	0.667	0.88 (0.65, 1.20)	0.417
		Q4	1.60 (1.18, 2.16)	0.002	1.29 (0.93, 1.78)	0.122	1.34 (0.97, 1.87)	0.081	1.21 (0.86, 1.71)	0.281

^a^Model1: unadjusted

^b^Model2: adjusted for age, SBP, DBP

^c^Model3: adjusted for age, SBP, DBP, TC, TG, smoking, drinking, use of antihypertensives, use of antilipidemic

^d^Model4: adjusted for age, SBP, DBP, TC, TG, smoking, drinking, use of antihypertensives, use of antilipidemic, haematologic, rheumatical, WBC, CRP, Hb

**Table 7:** Relationship between RAR and the risk of carotid plaques according to different glucose regulation state and age

Glucose regulation state	Age	Variables	Carotid artery plaques							
			OR (95% CI)^a^	*P*-value	OR (95% CI)^b^	*P*-value	OR (95% CI)^c^	*P*-value	OR (95% CI)^d^	*P*-value
Normal glucose regulation	< 60	RAR	1.46 (1.10, 1.94)	0.009	1.50 (1.10, 2.04)	0.011	1.55 (1.13, 2.13)	0.007	1.51 (1.06, 2.16)	0.022
		Q1	Reference		Reference		Reference		Reference	
		Q2	1.09 (0.80, 1.50)	0.578	1.09 (0.78, 1.53)	0.618	1.07 (0.76, 1.51)	0.695	1.06 (0.75, 1.51)	0.743
		Q3	1.11 (0.79, 1.55)	0.552	1.10 (0.77, 1.57)	0.612	1.08 (0.75, 1.56)	0.673	1.05 (0.72, 1.53)	0.808
		Q4	1.56 (1.09, 2.24)	0.016	1.63 (1.10, 2.40)	0.014	1.67 (1.12, 2.49)	0.011	1.62 (1.05, 2.49)	0.028
	≥ 60	RAR	1.40 (1.12, 1.76)	0.003	1.28 (1.01, 1.61)	0.040	1.23 (0.97, 1.57)	0.084	1.26 (0.97, 1.64)	0.080
		Q1	Reference		Reference		Reference		Reference	
		Q2	1.00 (0.76, 1.32)	0.999	1.02 (0.76, 1.37)	0.886	1.03 (0.77, 1.38)	0.847	1.03 (0.76, 1.38)	0.873
		Q3	1.15 (0.86, 1.53)	0.340	1.09 (0.81, 1.46)	0.576	1.06 (0.78, 1.44)	0.707	1.04 (0.77, 1.41)	0.796
		Q4	1.50 (1.13, 2.01)	0.006	1.41 (1.04, 1.90)	0.025	1.35 (0.99, 1.85)	0.056	1.38 (1.00, 1.91)	0.051
Prediabetes	< 60	RAR	1.05 (0.78, 1.42)	0.736	1.08 (0.79, 1.48)	0.645	1.10 (0.79, 1.53)	0.570	1.20 (0.81, 1.79)	0.372
		Q1	Reference		Reference		Reference		Reference	
		Q2	1.05 (0.73, 1.50)	0.812	1.01 (0.69, 1.49)	0.950	1.04 (0.71, 1.54)	0.827	1.08 (0.72, 1.61)	0.704
		Q3	0.83 (0.56, 1.23)	0.357	0.89 (0.59, 1.36)	0.599	0.90 (0.59, 1.37)	0.617	0.90 (0.59, 1.40)	0.651
		Q4	1.02 (0.66, 1.56)	0.947	1.04 (0.66, 1.64)	0.854	1.07 (0.67, 1.70)	0.793	1.20 (0.71, 2.04)	0.495
	≥ 60	RAR	1.22 (0.95, 1.56)	0.113	1.23 (0.95, 1.58)	0.119	1.22 (0.94, 1.59)	0.126	1.23 (0.92, 1.64)	0.163
		Q1	Reference		Reference		Reference		Reference	
		Q2	1.11 (0.82, 1.51)	0.511	1.19 (0.87, 1.64)	0.269	1.19 (0.87, 1.64)	0.273	1.20 (0.87, 1.65)	0.269
		Q3	1.35 (0.99, 1.84)	0.056	1.39 (1.01, 1.91)	0.041	1.35 (0.98, 1.86)	0.069	1.37 (0.99, 1.90)	0.056
		Q4	1.27 (0.94, 1.73)	0.124	1.34 (0.98, 1.84)	0.071	1.33 (0.96, 1.85)	0.083	1.33 (0.95, 1.87)	0.100
Diabetes	< 60	RAR	1.37 (1.04, 1.82)	0.027	1.45 (1.08, 1.96)	0.015	1.64 (1.20, 2.24)	0.002	1.59 (1.13, 2.24)	0.008
		Q1	Reference		Reference		Reference		Reference	
		Q2	1.26 (0.90, 1.76)	0.176	1.30 (0.91, 1.84)	0.145	1.31 (0.92, 1.87)	0.139	1.31 (0.92, 1.88)	0.140
		Q3	1.00 (0.71, 1.42)	0.998	1.01 (0.71, 1.46)	0.944	1.12 (0.78, 1.63)	0.537	1.09 (0.75, 1.59)	0.653
		Q4	1.70 (1.15, 2.52)	0.008	1.84 (1.22, 2.77)	0.004	2.16 (1.41, 3.30)	< 0.001	2.10 (1.33, 3.33)	0.002
	≥ 60	RAR	1.39 (1.11, 1.75)	0.005	1.43 (1.12, 1.81)	0.004	1.52 (1.19, 1.94)	0.001	1.35 (1.03, 1.76)	0.031
		Q1	Reference		Reference		Reference		Reference	
		Q2	0.83 (0.62, 1.12)	0.220	0.84 (0.62, 1.14)	0.260	0.83 (0.61, 1.13)	0.242	0.83 (0.61, 1.12)	0.219
		Q3	0.91 (0.69, 1.22)	0.535	0.95 (0.71, 1.27)	0.712	0.95 (0.71, 1.28)	0.740	0.91 (0.67, 1.23)	0.530
		Q4	1.31 (0.97, 1.78)	0.078	1.34 (0.98, 1.83)	0.065	1.39 (1.02, 1.91)	0.040	1.23 (0.88, 1.71)	0.219

^a^Model1: unadjusted

^b^Model2: adjusted for sex, SBP, DBP

^c^Model3: adjusted for sex, SBP, DBP, TC, TG, smoking, drinking, use of antihypertensives, use of antilipidemic

^d^Model4: adjusted for sex, SBP, DBP, TC, TG, smoking, drinking, use of antihypertensives, use of antilipidemic, haematologic, rheumatical, WBC, CRP, Hb

### Relationship between RAR and the number and echo characteristics of carotid plaques

As shown in Additional file 1: Table S2, when RAR was included as a categorical variable, RAR was significantly correlated with multiple carotid plaques, the Q4 group was 1.26 times higher than the Q1 group. As shown in Additional file 1: Table S3, when RAR was included as a continuous variable, after adjustment for confounding factors, RAR was significantly correlated with hypoechoic plaque (OR: 1.27; 95% CI 1.00–1.59), and mixture plaque (OR: 1.48; 95% CI 1.28–1.71). When RAR was included as a categorical variable, RAR was significantly associated with mixture plaque, the Q4 group was 1.66 times higher than the Q1 group.

## Discussion

Our study found that RAR was significantly related to carotid plaque formation in CHD patients and was strongest in diabetic glycemic state. To the best of our knowledge, this is the first study to investigate the predictive value of RAR, a novel combined biomarker, with carotid plaque in patients with CHD.

RDW reflects the size distribution of red blood cells. Traditionally, it is used together with other hematological indicators for the differential diagnosis of anemia. Research shows that RDW can be considered as an indicator of chronic inflammation [21]. In recent years, it has been gradually found that RDW is associated with CVD mortality [22, 23], RDW can also be used as a predictor of adverse outcomes in patients with carotid atherosclerosis [24]. High RDW correlates with an increased risk of carotid atherosclerosis [25]. In a cross-sectional study of middle-aged and elderly hypertensive patients, the results showed that with the increase of RDW, the proportion of carotid atherosclerosis patients increased [26]. In a cohort study, it was found that RDW was associated with plaque in carotid atherosclerosis, and the association between RDW and the development of CVD could be explained by atherosclerosis [27]. The erythrocyte membrane contains a large amount of free cholesterol [28], the accumulation of erythrocytes within the atherosclerotic plaque may promote plaque growth and instability [29]. Unstable plaques rich in cholesterol are easy to rupture, so prone to cause acute atherosclerosis events.

ALB is an essential protein in human plasma, widely used to evaluate the nutritional status and reflect the systemic information [30]. Hypoalbuminemia results from and reflects the inflammatory state [31]. Atherosclerosis is an inflammatory disease, which participates in the process of CVD [32]. ALB plays an anti-inflammatory role in this process, and once ALB decreases, the disease process will deteriorate. Several studies have reported an association between low ALB and an increased risk of cardiovascular events [33, 34]. In addition, the prognostic value of low ALB in acute coronary syndrome and stable CHD has also been reported [35–37]. The synthesis rate of ALB is affected by nutrient intake and systemic inflammation [38]. Moreover, the decrease of ALB level is related to the increase of blood viscosity, impairment of endothelial function, and the increase of synthesis of an important mediator of platelet-derived carotid stenosis [39–42]. These may be a potential mechanism to link serum ALB with the severity of carotid artery disease.

RAR is a combination of two classic clinical evaluation parameters, and RAR is a potential novel biomarker, which can be quickly and easily obtained in the laboratory tests on admission. In recent years, studies have shown that RAR is closely related to CHD, and it may be a key indicator for evaluating the severity of CVD. Li et al. found that RAR is a simple and a risk factor for poor prognosis in acute myocardial infarction (AMI) patients, the lower RAR level, the higher the short-term and long-term survival rate of patients [43]. Weng et al. believed that RAR had a better predictive ability for all-cause mortality of patients after PCI, and was superior to RDW or ALB alone. The RAR of patients with CHD may be positively correlated with the severity of CAD [44]. The rupture of atherosclerotic plaque and subsequent thrombosis are the main causes of acute cardiovascular events [27]. Our results suggest that the formation and development of carotid atherosclerosis may play a role in the relationship between RAR and cardiovascular incidence rate and mortality.

We also observed the relationship between RAR and carotid plaque by sex and age. RAR was associated with the risk of carotid plaque in female. And the relationship between RAR and carotid plaque was stronger in patients under 60 years of age than in those over 60 years of age. Similar results were found in a previous retrospective cohort study of the association of RAR in patients with AMI, the subgroup analysis showed that the effect of RAR was higher in female patients than in male patients [43]. We thought it might have something to do with women’s hormone levels. In the study on the influence of RDW and RAR on the all-cause mortality of patients with type 2 diabetes mellitus and foot ulcer, it was found that RAR was associated with high all-cause mortality of patients aged < 65 years old and patients aged ≥ 65 years old, but the association was stronger in younger and middle-aged patients [45], which was similar to our results.

Moreover, under different glucose metabolism states, RAR has the highest correlation with the risk of carotid plaque in diabetic blood glucose state. Studies have shown that RAR is strongly associated with all-cause mortality in diabetes [45], which may be because diabetes is related to changes in deformability and mechanical properties of red blood cells, increased adhesion and increased osmotic vulnerability [7]. Research shows that in the sample of adult diabetes patients, higher RDW values are associated with an increased risk of CVD [9]. It is known that chronic hyperglycemia accelerates atherosclerosis by increasing oxidative stress [46], which may be one of the potential mechanisms of increased RDW and increased risk of carotid plaque. Inflammation can affect erythropoiesis, red blood cell cycle half-life and red blood cell deformability, promote cell heterotopia, and thus improve the level of RDW [47]. Higher RDW levels are independently associated with higher CRP levels [48], which is a recognized marker of inflammation and CVD [49]. Diabetes is associated with chronic inflammation [50]. Inflammatory activity has been confirmed to increase in patients with type 2 diabetes. Similarly, the increase in the concentration of inflammatory markers (such as CRP) is also involved in the occurrence and progression of long-term macrovascular complications in diabetes [50]. Thus, the relationship between RDW and inflammation can at least partially explain the relationship between CVD and RDW that we found in diabetes patients.

At the same time, among the plaque echo property, RAR has the strongest relationship with mixture plaque. The hypoechoic plaque contains relatively more lipid components and more inflammatory substances, and the plaque are easy to rupture, with poor stability, while hyperechoic plaques have the opposite. RAR showed no obvious specificity between hyperechoic and isoechoic plaque properties.

This study was a multicenter, retrospective clinical study. To our knowledge, this is the first study on the correlation between RAR and carotid plaque in patients with CHD. This study has a large number of participants, and we have established several confounding factor models and adjusted them, and the results are convincing. However, this study has several limitations. First of all, this is a multi-center study, and there may be deviations in the measurement methods of different research centers. However, the quality of external clinical laboratories in each center is evaluated by practitioners, which greatly improves the reliability. Furthermore, body mass index (BMI) is an important confounding factor in CHD and carotid plaques. Because much BMI data were missing from this study, it was not included in the model. Finally, this is a cross-sectional study. It is difficult to draw a causal conclusion without taking time as a factor. We plan to further explore this relationship in a prospective study in future.

## Conclusion

Our study demonstrated that RAR in patients with CHD was significantly related to carotid plaque. This association was more significant in women and patients aged < 60 years people. In addition, under different glucose metabolism states, RAR has a higher correlation with the risk of carotid plaque in DM patients. In conclusion, RAR, as a simple and practical parameter, provided reference value for the prevention and risk stratification of carotid plaque in patients with CHD.

## Supplementary Information

**Additional file 1: Table S1.** Additive interaction between RAR and diabetes for the risk of carotid plaques. **Table S2.** Relationship between the RAR and the risk of number of carotid plaque. **Table S3.** Relationship between the RAR and the risk of carotid plaque echo property.

## Data Availability

The datasets used and/or analyzed in the present study are available from the corresponding author on reasonable request.

## Abbreviations

ALB: Serum albumin
CIs: Confidence intervals
CHD: Coronary heart disease
CVD: Cardiovascular disease
DBP: Diastolic blood pressures
DM: Diabetes
FPG: Fasting plasma glucose
HbA1c: Glycated hemoglobin
HDL-C: High-density lipoprotein-cholesterol
IMT: Intima-media thickness
LDL-C: Low-density lipoprotein-cholesterol
ORs: Odds ratios
Pre-DM: Prediabetes
RAR: Red cell distribution width/albumin ratio
RDW: Red cell distribution width
SBP: Systolic blood pressures
TC: Total cholesterol
TG: Triglyceride
