# Supplementary material for: Relationship between red cell distribution width/albumin ratio and carotid plaque in different glucose metabolic states in patients with coronary heart disease: a RCSCD-TCM study in China
Source: Cardiovasc Diabetol. 2023 Feb 22;22:39. doi: 10.1186/s12933-023-01768-w (PMC9948352; doi:10.1186/s12933-023-01768-w)
Supplement: Supplementary file 1 — Additional file 1: Table S1. Additive interaction between RAR and diabetes for the risk of carotid plaques. Table S2. Relationship between the RAR and the risk of number of carotid plaque. Table S3. Relationship between the RAR and the risk of carotid plaque echo property. [file 12933_2023_1768_MOESM1_ESM.docx]

**Additional file 1**

**Table S1** Additive interaction between RAR and diabetes for the risk of carotid plaques

| Joint exposure | | Carotid artery plaques | | | | | | | |
| --- | --- | --- | --- | --- | --- | --- | --- | --- | --- |
| Diabetes | RAR | OR (95% CI)^a^ | *P*-value | OR (95% CI)^b^ | *P*-value | OR (95% CI)^c^ | *P*-value | OR (95% CI)^d^ | *P*-value |
| No | Low | Reference |  | Reference |  | Reference |  | Reference |  |
| No | High | 1.47(1.29,1.69) | <0.001 | 1.22(1.05,1.41) | 0.008 | 1.24(1.07,1.44) | 0.005 | 1.21(1.04,1.41) | 0.016 |
| Yes | Low | 1.39(1.25,1.55) | <0.001 | 1.35(1.20,1.52) | <0.001 | 1.32(1.17,1.48) | <0.001 | 1.30(1.15,1.46) | <0.001 |
| Yes | High | 2.25(1.87,2.71) | <0.001 | 1.83(1.50,2.23) | <0.001 | 1.84(1.50,2.25) | <0.001 | 1.84(1.49,2.27) | <0.001 |

High RAR is RAR Q4 group; low RAR is the merge of RAR Q1, Q2, and Q3 group.

^a^Model1: unadjusted

^b^Model2: adjusted for sex, age, SBP, DBP

^c^Model3: adjusted for sex, age, SBP, DBP, TC, TG, smoking, drinking, use of antihypertensives, use of antilipidemic

^d^Model4: adjusted for sex, age, SBP, DBP, TC, TG, smoking, drinking, use of antihypertensives, use of antilipidemic, haematologic, rheumatical, WBC, CRP, Hb

**Table S2** Relationship between the RAR and the risk of number of carotid plaque

| Variables | Carotid plaque=1 (n=421) | | | | | | | |
| --- | --- | --- | --- | --- | --- | --- | --- | --- |
|  | OR (95% CI)^a^ | *P*-value | OR (95% CI)^b^ | *P*-value | OR (95% CI)^c^ | *P*-value | OR (95% CI)^d^ | *P*-value |
| RAR | 1.26(1.00,1.58) | 0.049 | 1.16(0.93,1.45) | 0.203 | 1.18(0.94,1.47) | 0.153 | 1.44(1.13,1.83) | 0.003 |
| Q1 | Reference |  | Reference |  | Reference |  | Reference |  |
| Q2 | 0.97(0.74,1.27) | 0.836 | 0.92(0.70,1.20) | 0.530 | 0.92(0.70,1.21) | 0.564 | 0.96(0.73,1.26) | 0.770 |
| Q3 | 0.71(0.53,0.95) | 0.023 | 0.64(0.47,0.87) | 0.004 | 0.65(0.48,0.89) | 0.006 | 0.69(0.51,0.93) | 0.017 |
| Q4 | 1.05(0.78,1.40) | 0.764 | 0.93(0.69,1.26) | 0.646 | 0.96(0.71,1.30) | 0.795 | 1.16(0.84,1.58) | 0.370 |
|  | Carotid plaque≥2 (n=7323) | | | | | | | |
| RAR | 1.54(1.39,1.71) | <0.001 | 1.22(1.09,1.36) | <0.001 | 1.26(1.12,1.41) | <0.001 | 1.21(1.07,1.37) | 0.003 |
| Q1 | Reference |  | Reference |  | Reference |  | Reference |  |
| Q2 | 1.13(1.00,1.28) | 0.050 | 0.98(0.86,1.12) | 0.790 | 0.99(0.86,1.13) | 0.828 | 0.98(0.85,1.12) | 0.755 |
| Q3 | 1.31(1.15,1.48) | <0.001 | 1.01(0.88,1.16) | 0.839 | 1.02(0.89,1.17) | 0.777 | 0.99(0.86,1.14) | 0.882 |
| Q4 | 1.75(1.54,2.00) | <0.001 | 1.27(1.10,1.47) | 0.001 | 1.31(1.13,1.51) | <0.001 | 1.26(1.07,1.47) | 0.004 |

^a^Model1: unadjusted

^b^Model2: adjusted for sex, age, SBP, DBP

^c^Model3: adjusted for sex, age, SBP, DBP, TC, TG, smoking, drinking, use of antihypertensives, use of antilipidemic

^d^Model4: adjusted for sex, age, SBP, DBP, TC, TG, smoking, drinking, use of antihypertensives, use of antilipidemic, haematologic, rheumatical, diabetes, WBC, CRP, Hb

**Table S3** Relationship between the RAR and the risk of carotid plaque echo property

| Variables | Hypoechoic plaque (n=512) | | | | | | | |
| --- | --- | --- | --- | --- | --- | --- | --- | --- |
|  | OR (95% CI)^a^ | *P*-value | OR (95% CI)^b^ | *P*-value | OR (95% CI)^b^ | *P*-value | OR (95% CI)^d^ | *P*-value |
| RAR | 1.40(1.14,1.71) | 0.001 | 1.22(1.00,1.49) | 0.055 | 1.29(1.05,1.58) | 0.016 | 1.27(1.00,1.59) | 0.046 |
| Q1 | Reference |  | Reference |  | Reference |  | Reference |  |
| Q2 | 1.14(0.87,1.49) | 0.337 | 1.06(0.81,1.39) | 0.697 | 1.06(0.81,1.39) | 0.668 | 1.06(0.80,1.39) | 0.702 |
| Q3 | 1.29(0.99,1.68) | 0.064 | 1.12(0.85,1.47) | 0.429 | 1.13(0.86,1.49) | 0.372 | 1.10(0.84,1.45) | 0.487 |
| Q4 | 1.49(1.13,1.96) | 0.005 | 1.26(0.95,1.67) | 0.109 | 1.33(1.00,1.77) | 0.054 | 1.28(0.94,1.73) | 0.113 |
|  | Isoechoic plaque (n=574) | | | | | | | |
| RAR | 1.24(1.02,1.52) | 0.035 | 1.10(0.90,1.34) | 0.368 | 1.11(0.91,1.36) | 0.294 | 1.11(0.88,1.38) | 0.386 |
| Q1 | Reference |  | Reference |  | Reference |  | Reference |  |
| Q2 | 1.01(0.79,1.29) | 0.954 | 0.95(0.74,1.22) | 0.683 | 0.95(0.74,1.22) | 0.675 | 0.94(0.73,1.21) | 0.648 |
| Q3 | 0.96(0.74,1.23) | 0.733 | 0.85(0.65,1.10) | 0.207 | 0.86(0.66,1.11) | 0.248 | 0.84(0.64,1.09) | 0.182 |
| Q4 | 1.18(0.91,1.53) | 0.214 | 1.01(0.78,1.32) | 0.926 | 1.03(0.79,1.36) | 0.815 | 1.02(0.76,1.36) | 0.902 |
|  | Hyperechoic plaque (n=4312) | | | | | | | |
| RAR | 1.48(1.32,1.66) | <0.001 | 1.17(1.04,1.31) | 0.011 | 1.18(1.05,1.33) | 0.006 | 1.12(0.98,1.28) | 0.097 |
| Q1 | Reference |  | Reference |  | Reference |  | Reference |  |
| Q2 | 1.10(0.96,1.26) | 0.159 | 0.94(0.82,1.09) | 0.424 | 0.94(0.81,1.09) | 0.422 | 0.93(0.81,1.08) | 0.365 |
| Q3 | 1.22(1.06,1.40) | 0.005 | 0.93(0.80,1.07) | 0.303 | 0.92(0.80,1.07) | 0.294 | 0.89(0.77,1.04) | 0.142 |
| Q4 | 1.61(1.39,1.85) | <0.001 | 1.14(0.98,1.33) | 0.097 | 1.15(0.98,1.35) | 0.083 | 1.10(0.93,1.29) | 0.286 |
|  | Mixture plaque (n=2346) | | | | | | | |
| RAR | 1.70(1.51,1.93) | <0.001 | 1.35(1.19,1.53) | <0.001 | 1.42(1.25,1.62) | <0.001 | 1.48(1.28,1.71) | <0.001 |
| Q1 | Reference |  | Reference |  | Reference |  |  |  |
| Q2 | 1.19(1.02,1.40) | 0.031 | 1.04(0.87,1.23) | 0.686 | 1.05(0.88,1.24) | 0.595 | 1.05(0.88,1.24) | 0.607 |
| Q3 | 1.45(1.24,1.70) | <0.001 | 1.12(0.95,1.33) | 0.186 | 1.14(0.96,1.36) | 0.128 | 1.13(0.95,1.34) | 0.186 |
| Q4 | 2.14(1.82,2.51) | <0.001 | 1.54(1.30,1.84) | <0.001 | 1.63(1.36,1.95) | <0.001 | 1.66(1.37,2.00) | <0.001 |

^a^Model1: unadjusted

^b^Model2: adjusted for sex, age, SBP, DBP

^c^Model3: adjusted for sex, age, SBP, DBP, TC, TG, smoking, drinking, use of antihypertensives, use of antilipidemic

^d^Model4: adjusted for sex, age, SBP, DBP, TC, TG, smoking, drinking, use of antihypertensives, use of antilipidemic, haematologic, rheumatical, diabetes, WBC, CRP, Hb
